# Behaviour Change in Public Health: Evidence and Implications

**DOI:** 10.1155/2015/598672

**Published:** 2015-08-26

**Authors:** Subhash Pokhrel, Nana K. Anokye, Daniel D. Reidpath, Pascale Allotey

**Affiliations:** ^1^Health Economics Research Group (HERG), Institute of Environment, Health and Societies, Brunel University London, Uxbridge UB8 3PH, UK; ^2^Public Health and SEACO, School of Medicine and Health Sciences, Monash University Malaysia, Jalan Lagoon Selatan, 47500 Bandar Sunway, Selangor Darul Ehsan, Malaysia

The evidence on the role of particular lifestyles, smoking, binge drinking, lack of physical activity, and poor health care seeking, in increased risks for mortality and morbidity is compelling [1]. Understanding the pathways through which these various “unhealthy” behaviours affect health is complicated by the broader ecological context in which they occur. The complexity is further enhanced because behaviours do not occur in isolation and there is often a convergence of associations. Interventions to achieve changes in either single or multiple behaviours have therefore often been limited in their effectiveness and longer term sustainability. In order to develop and implement a meaningful behaviour change agenda we need to establish innovative ways of operationalising and understanding the complexity of behavioural factors and their dynamic interrelationships and how these collectively affect health. The Behaviour Change Research Cycle (BCRC) (Figure 1) provides a simple illustration of the life cycle of evidence required.

Extant research on behaviour change interventions highlights the importance of structural support and a range of requisites such as capability, motivation, and opportunities [2]. Taking this broader perspective on behaviour change extends the field beyond merely a psychological enquiry aimed at understanding individuals' behaviour to a more pragmatic endeavour that includes research that can actively inform the development and implementation of interventions. Evaluation of the appropriateness, cost effectiveness, affordability, acceptability, and sustainability of the intervention follows thereafter. Generating evidence on behaviour change is therefore as diverse as the academic disciplines supporting wider public health practice [3].

Through this special issue, robust research on behaviour change, as envisaged and implemented across the range of public health disciplines, is presented to enhance our understanding of how behaviour change research could inform public health practice at local, regional, and global levels. The selected papers in this issue provide* avant-garde* research across the various levels of the behaviour change research cycle.

Research on* motivators of certain behaviours* is presented through three studies included in this special issue. W. Liang and T. Chikritzhs explore survey data and find a significant association between heavy alcohol use and risk of violence. Likewise, pro- and antitobacco imagery as seen in media and other outlets appear to be an important determinant of the attitudes towards and uptake of smoking among adolescents. G. Waqa et al. conclude therefore that public health warnings can work in real life. Most behaviour change (positive or negative) happens when individuals find themselves in a completely new context and this hypothesis is demonstrated in the study conducted with Filipino migrants by D. Maneze et al. where competing priorities of daily living were perceived by study participants as a key barrier to their health-seeking behaviour.

The* role of technology in supporting behaviour change* is increasingly important although the evidence remains sparse. In their systematic review, J. L. Watterson, et al. focus on the mobile health (mHealth) technologies in low- and middle-income settings to improve behaviours related to maternal and child health. They find some evidence of effectiveness in changing behaviour to improve antenatal/postnatal care attendance or childhood immunisation rates. Likewise, “nudging” through a smartphone application, SmartAPPetite, is the subject of a study by J. Gilliland et al. in which they find the application was effective in increasing a sense of improved awareness and consumption of healthy foods. The use of technology as a research tool is also an increasingly valuable innovation and is explored in the study by E. Bisung et al. using “photovoice.” The method employs the use of photography as an effective participatory research tool to understand behaviours, create awareness, and support sanitation and hygiene related behaviour change at community levels.

The real-life evidence presented in this special issue offers an interesting notion of “*exotic*”* public health practice*. It is exotic in the sense that implementing behaviour change can often cross the boundaries of national health services and this can happen at home, as demonstrated by E. L. Melbye and H. Hansen in relation to prevention focused feeding strategies, as well as at school classroom as investigated by M. Bronikowski et al. They test the hypothesis whether support given through teachers and peers can have positive effect on stimulating adolescents' physical activity levels.

Other real-life evaluations include studies by A. Gigantesco et al. on school-based mental health programme, by B. AM Schutte et al. on BeweegKuur lifestyle intervention implemented in Dutch primary healthcare settings, by H. Limm et al. on a health promotion program based on a train-the-trainer approach, and by L. H. Norton et al. on pedometer or instructor-led group protocol to increase physical activity levels. How socioeconomics can impact lifestyle risk factors is the subject of enquiry in the study by S. Streel et al. while P. Sedlak et al. present long term lifestyle changes in preschool children.

Finally, implementing behaviour change is replete with challenges not least of which is* stakeholder engagement*. A.-M. Hendriks et al. provide a policy analysis of local decision making in Fiji. Obesity prevention has been difficult to implement and major impediments include power inequalities across various actors due to lack of engagement. A. Linden provides an informative analysis of the missing data generated as the unintended consequence of lack of patient (or service users) engagement in public health research. Stakeholder engagement throughout and beyond the research process therefore seems inevitable for a successful behaviour change agenda.

*****Subhash**  **Pokhrel***** *****Nana**  **K.** **Anokye***** *****Daniel**  **D.**  **Reidpath***** *****Pascale**  **Allotey*****

## Figures and Tables

**Figure 1:**
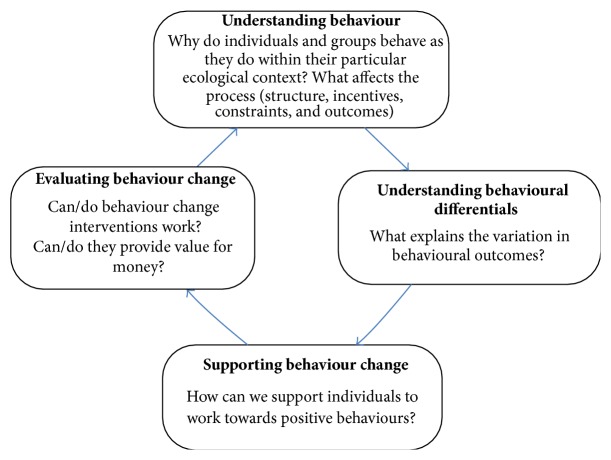
Behaviour Change Research Cycle (BCRC).
